# Childhood cancer and parental mental health: role of disease severity, socioeconomic status, and social dynamics

**DOI:** 10.1186/s12888-025-06494-z

**Published:** 2025-01-17

**Authors:** Hawkar Ibrahim, Azad Ali Ismail, Narin Ahmed Rahim, Benjamin Iffland, Frank Neuner

**Affiliations:** 1 Department of Psychology, Division of Clinical Psychology and Psychotherapy, Bielefeld University, P.O. Box 100131, Universitätsstraße 25, Bielefeld, 33501 Germany; 2 Department of Clinical Psychology, Koya University, Koya, Kurdistan Region Iraq; 3 Independent Researcher, Erbil, Kurdistan Region Iraq

**Keywords:** Childhood cancer, Parental mental health, Social constraints, Social dynamics, Socioeconomic impact

## Abstract

**Background:**

The impact of childhood cancer extends beyond the affected child, significantly influencing the mental health of their families. Since research in psycho-oncology has been carried out almost exclusively in high-income countries, little is known about the impact of childhood cancer on the family level in low- and middle income countries (LMICs). This is a notable gap in the evidence-base, as many LMICs are collectivist cultures, where social and family networks are crucial elements of health care.

**Methods:**

This prospective, cross-sectional study examined the mental health of 307 Kurdish parents of children undergoing cancer treatment in the Kurdistan Region of Iraq (KRI). Data were collected through structured interviews at two major oncology hospitals in the KRI, utilizing standardized instruments to assess mental health symptoms, socioeconomic status, and social constraints. The study focused on understanding the effects of socioeconomic challenges, disease severity, and social support limitations on parental mental health.

**Results:**

Significant mental health challenges were observed among parents, with mothers experiencing higher symptom levels than fathers. Lower socioeconomic status was linked to poorer parental mental health. Additionally, parents of children with more severe cancer and those reporting their child’s reduced engagement in daily activities experienced heightened mental health symptoms. Social constraints on expressing feelings about the child’s illness further intensified parental stress, underscoring the cumulative impact of economic strain, disease severity, and limited social support on parental mental health.

**Conclusions:**

The study highlights the intricate relationship between socioeconomic factors, the disease severity of the child, and social dynamics in shaping parental mental health in the context of pediatric cancer. Psychosocial interventions that target these specific stressors may aid in better supporting families, particularly those in low-resource settings.

## Introduction

Childhood cancer not only poses significant mental health risks for the affected child, but also has a significant impact on their caregivers, particularly parents [1, 2]. Studies have reported increased distress among caregivers of children with cancer [3, 4]. Such psychological distress encompasses increased rates of post-traumatic stress, depression, adjustment disorders, and anxiety [5–7]. Furthermore, research consistently showed that parents of children with cancer are at a higher risk of mental health challenges compared to the general population [6, 8–12].

Research is needed to explore the complex factors that influence the relationship between a childhood cancer diagnosis and reduced parental mental health [13, 14]. This examination requires an analytical lens that extends beyond the immediate psychological effects of the diagnosis to include a spectrum of contributing factors, ranging from the individual micro level to the macro level of family dynamics and policy influences. Thus, the current study adopts a socio-ecological framework to analyse the multiple determinants that shape parents’ psychological well-being following their child’s cancer diagnosis. This analytical framework allows for a nuanced examination of individual, interpersonal, and systemic dimensions, along with the child’s demographic and medical characteristics, to illuminate the complex interplay of factors affecting parents’ mental health.

At the individual level, it has been reported that parental role, as well as education and socioeconomic status, have a significant impact on the mental health of parents whose children have cancer. For instance, it has been reported that mothers and fathers were differentially affected by their child’s cancer diagnosis, with mothers’ being affected more severely [7, 11]. Greater levels of distress in mothers than in fathers have been related to higher care burdens on mothers [15]. Moreover, socioeconomic status, including financial hardship, unemployment, lower income, and lower educational level, represent key predictors of mental health outcomes in caregivers of patients with cancer [16, 17]. The substantial financial burden of a child’s cancer diagnosis and treatment (such as reduced work hours, job loss, and travel expenses) can intensify psychological distress, especially among parents with lower socioeconomic status. Notably, Ramsey and colleagues [18] found that a caregivers’ subjective feelings of financial difficulties, rather than objective markers of poverty, were associated with poorer mental health. In line with this, perceived financial difficulty has been identified as a prominent psychosocial risk factor for adjustment during diagnosis and treatment in families affected by cancer [19, 20]. In addition, parental educational level has been related to a better understanding of cancer and greater ability to manage symptoms and make medical decisions [21], which may alleviate psychological distress in parents of children with cancer. However, the extent to which socioeconomic factors influence the mental health of parents of children with cancer in low-resource contexts has not yet been investigated.

At the interpersonal level, unsupportive social conditions characterized by social constraints [22, 23] seem to facilitate a parent’s poor adjustment to their child’s cancer diagnosis. It has been well documented that disclosing thoughts, feelings, and concerns related to cancer to close ones improves emotional and cognitive processing and engages adaptive coping strategies by supporting contemplation and tolerance of cancer-related thoughts and concerns [23, 24]. However, parents of children with cancer often face social constraints that discourage and limit their expression of cancer-related thoughts and feelings [22]. These social constraints are associated with avoidance of thinking and talking about cancer [25–27] and, as a consequence, are related to poorer adjustment, heightened post-traumatic stress symptomatology, and higher depressive symptoms among caregivers and parents of patients with cancer [10, 28, 29]. So far, most research on the impact of negative social conditions such as social constraints on the psychological distress of cancer patients and their caregivers has been conducted in WEIRD (Western, Educated, Industrialized, Rich, and Democratic) countries. However, the effects of social constraints on mental health outcomes might be even more pronounced in community-oriented contexts. Therefore, the present study sought to examine the influence of social constraints on mental health in parents of children with cancer in a Kurdish sample. The Kurdish social fabric is known for its family-oriented and collectivist nature, with an emphasis on strong social ties and family networks [30, 31]. In this context, social support is expected. However, if close ones react with social constraints to the disclosure of thoughts, feelings, and concerns related to cancer instead of providing social support, this may lead to even more psychological distress and mental health problems.

Alongside individual, interpersonal, and community factors, several studies have highlighted the impact of the child’s medical factors on parental distress. Parental distress increased in advanced stages of cancer of their children reflecting higher illness severity, while it decreased as a function of duration since cancer diagnosis [7, 17]. In addition, parental distress differed with the type of treatment and treatment stage [6]. Moreover, the type of cancer and the associated impairments have an influence on parental adjustment to their child’s cancer [18, 23]. Hence, when investigating parents’ adjustment to their child’s cancer diagnosis, it is necessary to examine not only the effects of their own socio-demographic and unsupportive social dynamic such as the social constraints imposed by their social networks, but also the medical profile of their children, which may have an impact on their adjustment.

By analysing how socio-demographic variables such as parental role, education and socio-economic status interact with the dynamics of social constraints and child medical characteristics, this research aims to shed light on the complex interplay of factors contributing to parental psychological distress. Particular attention will be paid to the impact of social constraints for Kurdish people in the Kurdistan region of Iraq, to explore how this community-oriented context may influence mental health outcomes. This study aims to provide valuable insights for the targeted development of psychosocial interventions to better support parents in coping with a child’s cancer diagnosis.

## Methods

### Sample and procedure

This prospective, cross-sectional study analysed data from 307 Kurdish parents from the Kurdistan Region of Iraq (KRI), comprising 115 fathers and 192 mothers, who had a child diagnosed with cancer. The research was conducted at two major public oncology hospitals: Hiwa Hospital in the city of Sulaymaniyah and Nanakali Hospital in the city of Erbil, which specialise in the treatment and care of both paediatric and adult cancer patients.

The recruitment process was facilitated by hospital administrators. Staff members informed eligible parents about the study. Eligibility criteria included having a child diagnosed with cancer at least one month after their diagnosis, currently receiving treatment at either hospital, and being aged 18 years or older. Full details of the study objectives, procedures, and potential consequences were provided to enable parents to make an informed decision about their participation. Interested parents were then invited for a detailed discussion with the research team, emphasizing understanding and agreement with the consent forms and ensuring that their rights within the study were clear. Before conducting interviews, the interviewers obtained standardized informed consent verbally and documented informed consent for each participant. The verbal informed consent was chosen for two reasons: first, some of the parents were illiterate and could not write and read; secondly, in the local context, signing documents is often linked to bureaucratic processes of authoritarian governments, which could have led participants to be suspicious that their information might be used for non-research purposes [32]. A total of 309 parents met the inclusion criteria and were invited for face-to-face interviews. All agreed to participate, although two were unable to continue with the interview due to caring responsibilities.

All interviews were conducted by four local clinical psychologists, who received training and supervision from the first author. The interviewers underwent extensive training in various areas of psycho-oncology and research in low-resource settings. The training encompassed ethical research practices, cultural sensitivity, psychological first aid, understanding the psychological impact of cancer on patients and their families, and strategies for supporting families and caregivers. Additionally, they were trained in building constructive relationships with cancer care teams and in self-care techniques. The psychologists also received detailed instructions on the study materials to maintain consistency and quality in the data collection process.

To ensure that all participants have access to mental health support, we took a comprehensive approach. We provided detailed information about available mental health services, both within the participating hospitals and from external sources to all participants, regardless of their initial mental health status. Special attention was given to participants who showed signs of mental health challenges, identified either through high scores on mental health assessments or direct requests for assistance. These participants were promptly referred to specialised mental health services within the hospitals. Furthermore, our study established collaborations with local non-governmental organisations (NGOs) that specialise in mental health and psychosocial support for adults. As part of this collaboration, literate participants received an informative brochure. The brochure included essential information on identifying signs of mental health issues and seeking support, such as the addresses and contact details of relevant NGOs.

The study’s procedures and methodologies have been reviewed and approved by the Research Ethic Committee of Koya University in the KRI, the Ethics Committee of Bielefeld University in Germany, as well as Ministry of Health in the KRI.

### Instruments

#### Socio-demographic questionnaire

A socio-demographic questionnaire was developed specifically to capture the unique cultural and health context of the participants. The questionnaire was divided into two main sections. The first section collected basic demographic data from participants, including their parental role, age, and marital status. The second section focused on the socio-demographic details of the participants’ children. It included questions about the child’s gender, age, birth order, school attendance and the child’s ability to participate in daily activities compared to their healthy siblings. Furthermore, a comprehensive parent-report questionnaire was designed to collect detailed medical information about their child’s cancer status. This questionnaire focused on several key aspects, including the specific type of cancer diagnosed, the stage of the cancer and the duration since diagnosis, as well as the type of treatment and care provided, clearly categorised into inpatient and outpatient services.

#### Economic status questionnaire

Economic status was assessed based on both objective and subjective indicators. The objective assessment focused on several factors: the nature of participants’ occupations (i.e., homemakers, employed in the public or private sectors, or self-employed), the presence of a regular income—whether derived from salary or other benefits such as income generated from property rentals—and ownership of houses and cars. In parallel, the subjective dimension, termed “livelihood perception” was evaluated through individuals’ self-assessment of their economic positioning relative to their social networks. Participants evaluated their ability to satisfy fundamental needs in comparison to their peers and relatives, categorizing their livelihood status into one of three levels: high, medium, or low. This subjective measure aims to capture the participants’ personal perceptions and experiences of economic security and their sense of financial adequacy or inadequacy within their community context.

#### Mental health symptomology

The Hopkins Symptom Checklist 25 (HSCL-25) was used to assess the prevalence and severity of mental health symptomology [33, 34]. The HSCL-25 has been translated into Kurdish and has been used extensively to assess symptoms of depression in Kurdish war and genocide survivors in Iraq [30, 35–37]. The HSCL-25 consists of 25 items that assess common mental health symptoms including anxiety and psychosomatic symptoms (e.g., excessive worry, nervousness, fear, and physical manifestations such as trembling and sweating) and depressive symptoms (e.g., sadness, loss of interest, hopelessness, and changes in sleep and appetite patterns). Participants were asked to rate how much each symptom bothered them in the past week. The average score of which ranges from 1 (“not at all”) to 4 (“extremely”), was calculated by dividing the total score by the number of items. The internal consistency of the HSCL-25 was high, with McDonald’s ω = 0.92 (95% CI [0.91, 0.93]).

#### Social constraints

This study utilized the Social Constraints Scale (SCS), a 15-item unidimensional measure developed by Lepore and Ituarte [38], to examine the social constraints experienced by parents in relation to their child’s cancer. The SCS assesses the presence of an unresponsive social network that typically prevents the expression of stress-related thoughts and emotions, thereby fostering a tendency towards avoidance [23]. Previous research has mainly used the SCS to investigate the experiences of cancer survivors [39–43], with limited to no focus on the experiences of family members or caregivers. In this study, we modified the SCS to be used for parents of children diagnosed with cancer. The purpose of adapting the SCS was to gain a deeper insight into complex interpersonal dynamics that parents of children with cancer may face in social situations, especially when expressing their thoughts, feelings, and concerns about their child’s illness.

A backward translation method was used to translate the scale from English to the Kurdish Sorani dialect. The Kurdish adaptation was assessed for face validity by a panel of local clinical psychologists. Careful review was conducted on each item to ensure accurate wording and phrasing for cultural appropriateness. Several modifications were made during this review to better align with the caregiving relationships of parents whose child has been diagnosed with cancer, which are central to our study. For example, all phrases referring to cancer in relation to a person, such as “*your cancer*”, were revised to “*your child’s cancer*”. These changes were necessary because our study focused solely on parents as caregivers.

Consistent with the methodology used for other instruments in the study, we administered the SCS through face-to-face interviews. Participants were asked to reflect on how often family members or friends had made comments or behaved in relation to their child’s illness that they found upsetting. Responses were recorded on a five-point Likert scale, ranging from 1 (indicating ‘never’) to 5 (indicating ‘very often’). The internal consistency of the SCS was demonstrated with a McDonald’s ω of 0.77 (95% CI [0.73, 0.80]).

### Data analysis

Means (M) and standard deviations (SD) were used to represent continuous variables, while frequencies (N) and percentages (%) were used for categorical variables. Normality of the data was assessed using Shapiro–Wilk’s test, Kolmogorov–Smirnov’s test, with histograms, and normal Q-Q plots. The results indicate that all analyzed variables and their residuals met the normality assumptions. The internal consistencies of the scales were assessed using McDonald’s omega (ω), which is presented alongside 95% confidence intervals (CI). As a robust measure of internal consistency, McDonald’s ω serves as an alternative to Cronbach’s alpha [44]. Pearson’s correlation coefficient (Pearson’s *r*) was used to examine linear associations between variables. Differences in demographic, socioeconomic, and health status factors between two participant groups were assessed using an independent samples t-test. Analysis of Variance (ANOVA) was used to analyze differences across multiple groups in relation to an independent variable on an ordinal scale. Hierarchical linear regression analysis was used to explore predictors of mental health symptomology. Only variables with a significant zero-order correlation with parental mental health were included in the regression model.

## Results

### Socioeconomic characteristics of participants and their children

The participants’ ages ranged from 23 to 46 years, with a mean age of 37.79 years (*SD* = 5.86). The vast majority (97.4%) were currently in a formal and legal marital relationship. On average, participants had 3.46 biological children (*SD* = 1.53). Economically, 66.8% (*n* = 205) indicated they lacked a regular income. This economic instability is notable when compared to the broader context of Iraq, where the national unemployment rate was 15.5% in 2023 according to the International Labour Organization [45]. In terms of livelihood perception, 41.7% classified themselves as low, 37.8% as middle, and 20.5% as high levels. Table 1. presents detailed information on the participants’ demographics, socioeconomic status, and health.

**Table 1:** Comprehensive participant profile: demographic, socioeconomic, and health status

Demographic Information		
Parental Roles, n (%)	Father	115 (37.5)
	Mother	192 (62.5)
Marital Status	Married	229 (97.4
	Divorce	3(1)
	Separated	4(1.3)
	Widow	1(.3)
Age, mean (SD)^a,b^		37.79(5.86)
Formal education, mean (SD)^a,c^		7.96 (5.61)
Number of children, mean (SD)^d^		3.46 (1.53)
Socioeconomic Information		
Occupation, n (%)	Homemaker	171(55.7)
	Employed in the Public Sector	87 (28.3)
	Self-Employment	49 (16)
Having regular income, n (%)	Yes	102(33.2)
	No	205(66.8)
Livelihood perception, n (%)	Low	128 (41.7)
	Middle	116 (37.8)
	High	63 (20.5)
House ownership, n (%)	Yes	199(64.8)
	No	108(35.2)
Car ownership, n (%)	Yes	183(59.6)
	No	124(40.4)
Children and self-Health Status		
Presence of Clinically Diagnosed Physical Illness, n (%)	Yes	52 (16.9)
	No	255(83.1)
Presence of Clinically Diagnosed Mental Illness, n (%)	Yes	16 (5.2)
	No	291 (94.8)
Presence of Another Child with Chronic Illness, n (%)	Yes	11 (3.6)
	No	296 (96.4)

^a^in years

^b^score range: 23–46

^c^score range: 0–24

^d^score range: 1–9

The participants reported that their children diagnosed with cancer had a mean age of 7.60 years (*SD* = 3.27), ranging from 1 to 16 years. Of these children, 32.6% were of kindergarten age, 13.4% were not enrolled in school due to the effects of cancer, and 54.1% were attending school. Regarding medical care, 78.2% were receiving outpatient treatment at the time of data collection, while 21.8% were inpatients. Leukaemia was the most commonly diagnosed cancer among the children, accounting for 59.9% of cases. Brain tumours and osteosarcoma were the next most frequent diagnoses, representing 13.7% and 7.8% of cases, respectively. A significant proportion of the children were in the early stages of cancer, with 24.1% in Stage I and 37.5% in Stage II. Treatment modalities varied: 58.3% of the children received chemotherapy, 2.3% underwent surgery, and 38.4% received a combination of both treatments. Table 2 presents a comprehensive demographic and medical profile of the children as reported by their parents.

**Table 2:** Parental reporting of children’s demographic and medical profiles

Demographic Information		
Child’s sex, n (%)	Boy	182 (59.3)
	Girl	125 (40.7)
Age, mean (SD)^a b^		7.60(3.27)
Birth order, n (%)	Firstborn	74(24.1)
	Second-born	92(30)
	Third-born	71(23.1)
	Fourth-born	31(10.1)
	Fifth-born	21(6.8)
	Sixth-born	12(3.9)
	Seventh to ninth-born	6(2)
School attendance, n (%)	Below school age	100(32.6)
	Not enrolled	41(13.4)
	Attending school	166(54.1)
Health Status		
Patient care, n (%)	Outpatient	240(78.2)
	Inpatient	67(21.8)
Child’s knowledge about their cancer diagnosis, n (%)	Yes	87(23.3)
	No	196(63.8)
	Somehow	24(7.8)
Types cancer, n (%)	Leukemia	184(59.9)
	Brain tumor	42(13.7)
	Osteosarcoma	24(7.8)
	Hepatoblastoma	7(2.3)
	Neuroblastoma	10(3.3)
	Lymphoma	21(6.8)
	Retinoblastoma	4(1.3)
	Kidney Cancer	12(3.9)
	Other	3(1)
Child’s cancer stage, n (%)	Lack of information	16(5.2)
	Stage I	74(24.1)
	Stage II	115(37.5)
	Stage III	45(14.7)
	Stage IV	57(18.6)
Cancer treatments that child received, n (%)	Chemotherapy	179(58.3)
	Surgery	7(2.3)
	Surgery and Chemotherapy	118(38.4)
	Other	3 (1)

^a^in years

^b^score range: 1–16

### Mental health symptomatology and socioeconomic status of parents

The mean score for mental health symptomology as measured by the HSCL-25, was 2.32 (*SD* = 0.71; score range = 1- 3.88). Mothers displayed significantly higher levels of mental health symptomology compared to fathers (Mothers: *M* = 2.55, *SD* = 0.64; fathers: *M* = 1.94, *SD* = 0.65) (*t* (305) = 7.945, *p* < 0.001). Furthermore, younger parental age was associated with higher levels of mental health symptoms (*r* = -0.12, *p* < 0.05). Parents who had more years of formal education exhibited lower levels of mental health symptoms (*r* = -0.22, *p* < 0.001).

Regarding their economic status, participants with a regular income reported significantly lower levels of mental health symptoms (*t* (305) = 4.684, *p* < 0.001). Higher regular income was significantly linked to lower levels of mental health symptoms (*r* = -0.27, *p* < 0.001). Parents’ livelihood perception was examined in relation to their mental health symptom scores. A one-way ANOVA revealed a significant effect of livelihood perception levels on psychopathology in parents of cancer patients, (*F* (2, 304) = 22.46, *p* < 0.001), with an effect size of η^2^ = 0.129. Post hoc comparisons using Tukey HSD tests indicated significant differences in psychopathology between parents with low and medium (*p* < 0.001) and low and high livelihood perception levels (*p* < 0.001). No significant difference was observed between medium and high livelihood perception levels (*p* > 0.05). This pattern highlights a significant association between lower levels of perceived livelihood and higher levels of parental mental health symptoms.

In terms of mental and physical health, there was no significant difference between parents with a clinically diagnosed physical illness and those without in reporting mental health symptoms (*t* (305) = 1.594, *p* > 0.05. However, parents who reported the presence of a clinically diagnosed mental health condition significantly scored higher on the HSCL-25 (*t* (305) = 2.910, *p* < 0.01). Parents who did not have any other chronically ill children at home had lower levels of mental health symptoms (*t* (305) = 1.880, *p* < 0.05).

### Parental mental health symptomatology and child’s characteristics

Parental mental health symptomatology was not found to be associated with sex, birth order, school attendance or age of a child diagnosed with cancer. A higher stage of the child’s cancer was associated with higher levels of parental mental health symptoms (*r* = 0.19, *p* < 0.001). To further investigate the psychological states of parents across different stages of their child’s cancer, a one-way ANOVA were carried out. The results indicated that there are significant differences in mental health symptoms (*F* (4, 302) = 4.093, *p* < 0.01) across all cancer stages, including the group of parents who did not know their child’s cancer stage. Further examination using pairwise comparisons confirmed notable differences between certain cancer stages. In particular, there were significant differences in mental health symptoms between stage I and stage IV (*p* < 0.01) and between stage I and stage III (*p* < 0.01). Other comparisons did not reach statistical significance. The homogeneous subsets analysis suggested that parents in Stage I formed a distinct group with the lowest psychopathology scores, while there were no significant differences among parents in the other stages. Parents whose children were receiving inpatient care for cancer reported higher levels of mental health symptoms compared to parents of children receiving outpatient care (*t* (305) = -3.227, *p* < 0.001).

No differences were found between the parents’ mental health and the type of treatment their child had received, nor in the length of time their child had been diagnosed with cancer (*p*s > 0.05). Moreover, parental mental health symptoms were not associated with whether the child was aware of their cancer diagnosis or not. Parents who reported that their child were able to participate in daily activities similar to their healthy siblings reported fewer mental health symptoms (*r* = -0.19, *p* < 0.001).

### Social constraints

**Fig. 1:**
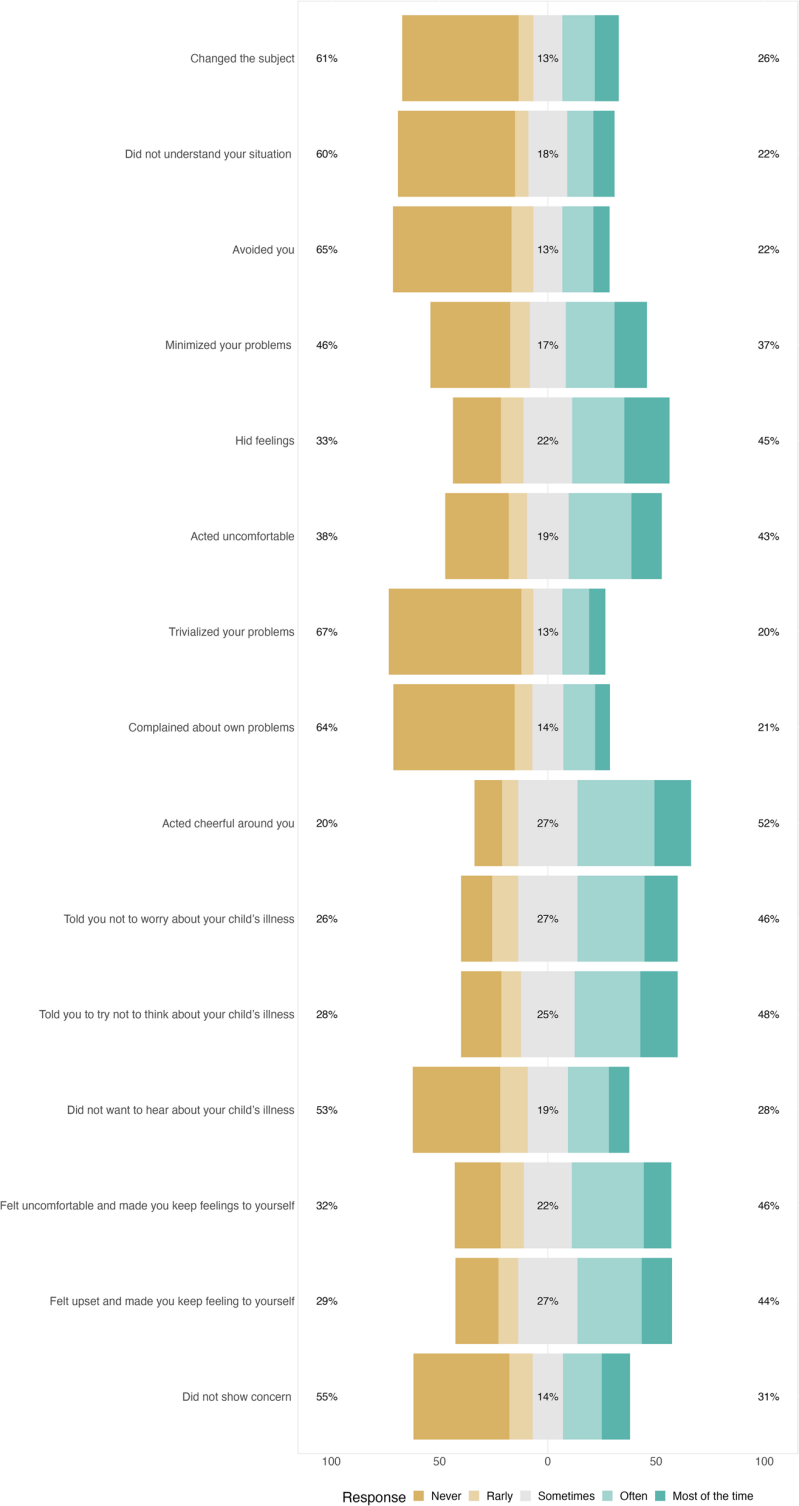
Likert plot analysis of responses to the social constraints scale

The SCS had a mean score of 40.07 (*SD* = 10.77). The range of scores was from 15 to 67, which is within the expectation range of 15 to 75. Figure 1 displays a Likert plot that illustrates the percentage distribution for each item on the SCS, thereby shedding light on the social barriers encountered by parents of children with cancer. The most prevalent social constraints, as reported “often” or “most of the time,”were “*trivialisation of problems*” (67%) and “*avoidance by others*” (65%). Furthermore, 61% of parents reported that others “*changed the subject*” when cancer-related issues arose, and 64% noted that others “*complained about their own problems*” indicating a lack of empathic engagement that may undermine social support.

A statistically significant negative correlation was found between the level of parental education and SCS scores. In particular, parents with lower levels of education reported higher SCS scores (*r* = -0.15, *p* < 0.01). Furthermore, a significant difference in reported social constraints was found between children’s treatment settings; parents of children receiving outpatient care reported higher levels of social constraints (*M* = 40.82) compared to parents of children receiving inpatient care (*M* = 37.35, *p* < 0.01). No significant association was found between SCS scores and factors such as parental role, age, regular income, or livelihood perception levels (*p*s > 0.05). Similarly, the length of time since their children were diagnosed with cancer and the cancer stage did not significantly correlate with SCS scores.

#### Predictors of mental health symptomatology

A hierarchical regression analysis was conducted to identify predictors of mental health symptomatology among parents. The analysis proceeded in three steps. The first step included socio-economic factors such as parental role, age, education, and perceived livelihood levels. This initial model accounted for 24.1% of the variance in mental health symptomatology (*R*2 = 0.241, *F* (4, 302) = 23.93, *p* < 0.001). The second step involved adding variables related to the child’s medical profile, such as cancer stage, participation in daily activities, and type of care received. This increased the explained variance to 28.5% (*R*2 = 0.285, *F* (7, 299) = 17.05, *p* < 0.001). The final model included the SCS, which further increased the explained variance to 31.8% (*R*2 = 0.318, *F* (8, 298) = 17.40, *p* < 0.001).

Table 3 summarizes the key predictors of poor parental mental health, including the mother’s parental role, lower livelihood levels, a higher cancer stage of the child, and experiencing more social constraints.

**Table 3:** Multiple hierarchical regression analysis predicting mental health symptomology

Factors		B	95% CI for B		SE B	β	R^2^	ΔR^2^	Zero-order correlation
			LL	UL					
Step I							.255		
	Parental role—mother	12.67^***^	8.9	16.44	1.91	.34			.41^***^
	Age	-.16	-.47	.15	.16	-.05			-.12^*^
	Education	-.23	-.58	.12	.18	-.07			-.22^***^
	Livelihood perception	-5.98^***^	-8.50	-3.45	1.28	-.25			-.35^***^
Step II							.30	.045	
Inclusion of	Cancer stage	2.10^**^	.59	3.62	.76	.13			.19^***^
	Participation of child in daily activities	-2.15^**^	-3.63	-.66	.75	-.14			-.19^***^
	Care type- outpatient	-2.26	-6.54	2.02	2.17	-.05			-.18^***^
Step III							.332	.032	
Inclusion of	Social constraints	.30^***^	.14	.46	.08	.18			.23^***^

*CI* confidence interval, *LL* lower limit, *UL* upper

^* **^p < .01

^***^p < .001

## Discussion

The present study examined the psychosocial experiences of parents of children diagnosed with cancer, aiming to understand the impact of the child’s medical profile and the parents’ socioeconomic and social status on parental mental health. The study revealed a significant link between parental mental health symptoms and several factors, including both parental socioeconomic status and child characteristics. The exploration of these relationships in a low-resource setting such as the KRI provides valuable insights into how limited resources and unique cultural factors influence the psychosocial experiences of parents dealing with their child’s cancer diagnosis.

The diagnosis of life-threatening medical conditions, such as paediatric cancer, is a critical event that can cause chronic stress and significantly affect the psychological well-being of parents. The higher levels of mental health symptoms reported by parents in this study are consistent with several systematic reviews and meta-analyses of the impact of childhood cancer on caregivers, particularly parents. For instance, van Warmerdam and colleagues [6] conducted a comprehensive meta-analysis to investigate the psychological burden on parents of children with cancer. The findings revealed that these parents are significantly more likely to experience psychological problems, especially anxiety, depression, and post-traumatic stress disorder, with more than 20% of them being affected by these conditions. In their meta-analysis, which included 35 studies with 11,396 participants from diverse cultural and socio-demographic populations, Bedaso et al. [46] found that globally, approximately two in five caregivers of cancer patients screened positive for major depressive disorder. Higher rates of mental health problems among parents of children diagnosed with cancer can be attributed to both the profound emotional distress and practical challenges associated with the child’s illness. Emotionally, parents often struggle with intense anxiety, feelings of helplessness and uncertainty about their child’s long-term health outcomes. The critical nature of the illness, coupled with the inherent uncertainty of treatment outcomes, contributes significantly to this distress [47–49]. On a practical level, the demanding role for the caregiver, which includes managing complex treatment plans and adapting to disrupted family dynamics and personal responsibilities, adds to the psychological burden on parents [50]. The combination of emotional and practical challenges leads to increased physical and psychological exhaustion, which confers risks to the mental health of these individuals.

Four major factors contributing to poor parental mental health in this study warrant attention: parental role, livelihood perception levels, child’s cancer severity, and social constraints.

Our results showed that mothers reported significantly higher levels of mental health symptoms compared to fathers, suggesting potential differences in the experience of caregiver distress within the parental dynamic. This observation is consistent with a systematic review that examined factors and consequences of parental distress associated with childhood cancer, which found that mothers generally report higher levels of distress compared to fathers [51]. In Kurdish culture, as well as in diverse global contexts, the role of the primary caregiver is frequently assumed by mothers, and this role presents a distinct set of challenges that can have significant implications for their mental health. Moreover, the scope of motherhood extends beyond caregiving tasks, particularly when caring for a child diagnosed with cancer. It encompasses a complex array of responsibilities, including balancing caregiving demands with household duties and employment obligations. The overlap of these responsibilities can increase stress levels and lead to mental health concerns in mothers. Furthermore, maternal caregiving stress is intensified by certain logistical policies implemented in pediatric units and hospitals within the KRI. In pediatric oncology units and children’s hospitals in the KRI, specific regulations add to the burden on mothers. These policies require that only mothers, or under exceptional circumstances other female relatives such as older sisters, aunts, or grandmothers, are authorized to act as caregivers for hospitalized children. The implementation of such regulations places a significant burden on mothers, especially those who do not have access to additional adult female support. This restriction becomes even more pronounced during extended hospital stays for cancer treatment, further limiting the ability of these mothers to seek and receive broader support.

Economic status was found to be a significant factor in predicting parental mental health symptomatology. In particular, participants with no regular income and those with a lower level of livelihood reported higher levels of mental health symptoms. This finding is consistent with previous research suggesting that economic hardship and financial stress contribute to higher levels of mental health symptoms among parents of children with cancer [52, 53]. It is well known that childhood cancer can have a significant financial impact on all families, but this burden is particularly pronounced for families with lower income levels [54–56]. Parents of children with cancer often struggle with the high costs associated with pediatric cancer treatment, including the expenses related to travel (i.e., taxi fares, car rentals, fuel consumption, and parking fees), as well as accommodation, food, and the purchase of essential medicines and supplies for their children’s cancer treatment [55, 57, 58]. These financial pressures could potentially lead to heightened levels of anxiety, depression, and overall psychological distress among parents. In the specific context it is important to consider that both Hiwa and Nanakali hospitals are government-funded health facilities that provide free-care and treatment to all cancer patients. However, due to ongoing political tensions between the central government of Iraq and the Kurdistan Regional Government (KRG), the Iraqi Ministry of Health does not regularly provide KRG oncology hospitals with essential medicines and oncology supplies. Consequently, patients and their families often bear the financial burden of procuring essential medicines and supplies out of their own pockets. However, while the current study did not assess the financial cost of childhood cancer to parents; during the interview, many parents expressed distress when asked about their economic status in managing their child’s treatment, medications and supplies. This challenge is compounded by the economic hardships faced by the Population in KRI, due to a financial crisis caused by the warfare of terrorist groups such as the Islamic State and the political instability and political tensions between the federal government of Iraq and the KRG.

This study identified two key factors related to a child’s illness that also predicted poor parental mental health: the severity of the child’s cancer and the diagnosed child’s ability to perform daily activities.

The finding that parents of children with advanced cancer stages report significantly higher levels of mental health symptoms (e.g., stage I vs. stage IV, *p* < 0.01) reinforces the profound impact of disease progression on caregiver distress. Advanced cancer often requires intensive treatments such as chemotherapy, radiotherapy, and surgery, which can lead to serious side effects and require prolonged care. These treatments and their associated side effects can significantly contribute to parental worry, exacerbating their psychological distress. In addition, the ability of children with cancer to engage in normal activities, including playing and interacting with their peers and siblings, also has a significant impact on parents’ psychological well-being. When a child can maintain some level of normalcy and engage in enjoyable activities, it can increase parents’ optimism and decrease their distress. In contrast, parents may become increasingly concerned about their child’s immediate and long-term well-being as they witness their child’s reduced ability to engage in usual activities or cope with treatment side effects. This concern often leads to heightened feelings of distress, worry, and anxiety.

The findings of this study suggest that social constraints are a significant risk factor for heightened mental health symptoms among parents of children with cancer. Hierarchical regression analysis confirmed that social constraints serve as a robust predictor of parental mental health outcomes, with the final model explaining 31.8% of the variance in symptom severity (R^2^ = 0.318, *F* (8, 298) = 17.40, *p* < 0.001). Prior research has not extensively focused on the role of social constraints on these parents. Manne et al., [29] have examined this issue, finding that social constraints are associated with higher post-traumatic stress symptomatology among mothers of children who had completed cancer treatment. Nonetheless, these findings align with the broader literature on social constraints, which indicates a significant impact on the psychological well-being of cancer survivors [59, 60].

The impact of social constraints on the mental health of parents with a child diagnosed with cancer can be explained through the theoretical framework of Lepore and Revenson [23]. The framework suggests that social constraints can lead to increased avoidance of thinking and talking about cancer, which can result in prolonged intrusive thoughts and psychological distress. Furthermore, this avoidance limits opportunities for parents to become familiar with cancer-related issues, gain new insights from others, and process and understand the cancer treatment path. Additionally, according to Lepore and Revenson when significant others engage in socially constraining behaviors, it can be particularly distressing, as such behaviors may conflict with parents’ expectations about the nature of their relationships and may undermine their sense of connection, trust, and safety.

In social contexts characterized by deeply rooted family-oriented values and close social networks, such as the Kurdish culture [31], the impact of social constraints may be more severe. In these social contexts, parents anticipate significant support from both their family and broader social networks. When they perceive, or are confronted with, social constraints, it may cause a strong contrast with their relationship expectations, thereby increasing their emotional and psychological distress [23]. Research within the Kurdish community has highlighted the importance of social networks, revealing that family and community interactions, particularly those involving social acknowledgment and social rejection, play a critical role in shaping the mental health of Kurdish individuals [30, 37]

The present study has several strengths that significantly contribute to the field of psycho-oncology. Firstly, it provides a unique focus on the psychosocial and economic challenges faced by parents in low- and middle-income countries. This perspective fills a crucial gap in the current literature, which predominantly centers on high-income Western regions. Second, the present study contributes significantly to the field of psych-oncology by examining the social constraints experienced by parents who are actively involved in the care of a child with cancer. Previous research on social constraints in the cancer context has focused on survivors or caregivers of children after treatment, so the current study provides a more comprehensive understanding of the caregiver experience during active treatment. Finally, the study’s thorough examination of parental socioeconomic factors and the demographic and medical profile of children with cancer allows for a detailed exploration of how these variables interact. This approach offers a multifaceted perspective on the complex dynamics between child health variables and caregiver psychological well-being, providing practical insights that can inform interventions and support mechanisms in psycho-oncology.

Despite its strengths, this study is not without limitations. The cross-sectional design limits the ability to establish causality and track changes in parental mental health over time. Additionally, the absence of a control group of parents with healthy children prevents the ability to identify the specific impact of childhood cancer on parental mental health. Furthermore, the economic assessment does not directly measure the financial burden of cancer treatment, which could have a significant impact on parental mental health. Additionally, the findings may be influenced by unmeasured variables, such as cultural attitudes towards mental health, which may affect both the experience and reporting of psychological symptoms. Acknowledging these factors as possible confounding variables provides a more comprehensive interpretation of the findings and highlights areas for further research.

To improve our understanding of parental mental health in the context of childhood cancer, future research in psycho-oncology should prioritize longitudinal study designs. These designs will allow for a more comprehensive exploration of how parental mental health changes over time and how social dynamics related to support and constraints change throughout the child’s treatment. Conducting studies in diverse cultural and regional contexts, both within the Middle East and globally, is crucial for understanding the role of sociocontextual factors in shaping the mental health of families of cancer survivors.

Given the unique challenges faced in low-income and resource-poor contexts, such as the KRI, where public health insurance is not yet available and the region is struggling with the long-term effects of war and political conflict, a thorough assessment of the financial impact of childhood cancer on families is urgently needed. This assessment should aim to understand the financial burdens faced by these families and how these economic challenges interact with mental health outcomes. Additionally, there is a significant/substantial need for intervention studies.that support parents of children with cancer. These interventions should be designed with careful consideration of sociocontextual factors and financial resources to ensure that they are accessible and effective in diverse settings. Interventions could specifically focus on providing psychological support, financial guidance, and community-based resources to assist parents in navigating the complexities of caring for a child with cancer.

Our research findings indicate a pressing need for the provision of psycho-oncology services in KRI oncology settings. It is recommended that the KRI’s policymakers and the KRG’s Ministry of Health consider implementing psycho-oncology services for cancer survivors and their families. The implementation of such services could assist in addressing the emotional and psychological needs of both cancer patients and their families, thereby improving the overall quality of cancer care and support in the region.

## Conclusion

The study highlights the complex relationship between socioeconomic factors, child health variables, and social dynamics in influencing the mental health of parents of children with cancer. It provides sociocultural insights that underline the need for targeted interventions and policy reforms to provide comprehensive support to these families.

## Data Availability

The datasets generated and/or analyzed during the current study are not publicly available due to the terms of consent agreed upon by the participants. However, they are available from the corresponding author upon reasonable request.

## Abbreviations

HSCL-25: The Hopkins Symptom Checklist 25
KRG: Kurdistan Regional Government
KRI: Kurdistan Region of Iraq
NGO: Non-governmental organisations
SCS: Social Constraints Scale
